# Nanotechnology as an Anti-Infection Strategy in Periprosthetic Joint Infections (PJI)

**DOI:** 10.3390/tropicalmed6020091

**Published:** 2021-05-28

**Authors:** Pier Francesco Indelli, Stefano Ghirardelli, Ferdinando Iannotti, Alessia Maria Indelli, Gennaro Pipino

**Affiliations:** 1 Department of Orthopaedic Surgery and Bioengineering, Stanford University School of Medicine, Stanford, CA 94305, USA; ghirardelli.stefano@gmail.com (S.G.); alessiaindelli@gmail.com (A.M.I.); 2 Department of Surgical and Medical Sciences and Translational Medicine, Sapienza University of Rome, 00185 Rome, Italy; ferdinandoiannotti@gmail.com; 3 Division of Orthopaedic Surgery, Vita-Salute San Raffaele University, 20132 Milan, Italy; dottgennaropipino@yahoo.it

**Keywords:** PJI, total knee arthroplasty, total hip arthroplasty, infection, nanotechnology, periprosthetic joint infections, diagnosis, nanoparticles, local delivery, antibiotic-loaded bone cement

## Abstract

Background: Periprosthetic joint infection (PJI) represents a devastating consequence of total joint arthroplasty (TJA) because of its high morbidity and its high impact on patient quality of life. The lack of standardized preventive and treatment strategies is a major challenge for arthroplasty surgeons. The purpose of this article was to explore the potential and future uses of nanotechnology as a tool for the prevention and treatment of PJI. Methods: Multiple review articles from the PubMed, Scopus and Google Scholar databases were reviewed in order to establish the current efficacy of nanotechnology in PJI preventive or therapeutic scenarios. Results: As a prevention tool, anti-biofilm implants equipped with nanoparticles (silver, silk fibroin, poly nanofibers, nanophase selenium) have shown promising antibacterial functionality. As a therapeutic tool, drug-loaded nanomolecules have been created and a wide variety of carrier materials (chitosan, titanium, calcium phosphate) have shown precise drug targeting and efficient control of drug release. Other nanotechnology-based antibiotic carriers (lipid nanoparticles, silica, clay nanotubes), when added to common bone cements, enhanced prolonged drug delivery, making this technology promising for the creation of antibiotic-added cement joint spacers. Conclusion: Although still in its infancy, nanotechnology has the potential to revolutionize prevention and treatment protocols of PJI. Nevertheless, extensive basic science and clinical research will be needed to investigate the potential toxicities of nanoparticles.

## 1. Background

Since total joint arthroplasty (TJA) has become a widespread procedure to treat degenerative joint disease, there has been a dramatic increase in the number of periprosthetic joint infections (PJI), a complication with high morbidity and mortality [1]. Costs associated with the treatment of PJI are double those associated with other, non-infection-related complications [2]; in fact, the economic burden of PJI has exceeded USD 1.62 billion in the USA alone. Recently, the current authors proposed a PJI preventive protocol [3] and a new surgical technique to retain an infected TJA implant [4]. Nevertheless, a broad consensus exists on the fact that the isolation or identification of the causative microorganism represents the main determinant for success in PJI treatment [5].

Nanotechnology utilizes the biological and structural properties of materials altered at the nanoscale (1–100 nm). Nanomaterials possess multiple physicochemical properties ideal for tissue and cell penetration and improvement in the resistance to static and dynamic fatigue [6,7,8]. Orthopedic surgeons recently increased their interest in this aspect of “nanomedicine” because of the multiple clinical applications, including implantable materials, targeted drug delivery and, ultimately, tissue regeneration. Specifically, it has been shown [9] that, once the size of particulate matter is diminished, the electrical and mechanical properties of many materials are modified; the final result of this process is represented by an improved interaction with the surrounding environment, which is a highly desired phenomenon in orthopedic surgery. In addition, the creation of drug-loaded nanomolecules has allowed for antibiotic therapy to directly target the microorganisms which routinely form the biofilm and infect orthopedic implants. 

In contrast to other reviews on the application of nanotechnology in orthopedics, our review focuses on the clinical aspects of nanotechnology, as a preventive and treatment strategy, in the subspecialty of musculoskeletal infections in general and PJI in particular. The current, clinical impact of nanotechnology in PJI prevention and treatment is very limited but the future potential is extremely appealing. 

## 2. Periprosthetic Joint Infection (PJI) Prevention

### 2.1. Implant Material

PJI involves bacterial colonization of the implant surface. Thus, any strategy to prevent bacterial colonization or biofilm formation should be focused on facilitating the engagement of host cells (fibroblasts, macrophages) and host proteins with the implant surface [10]. It has been shown that biofilms develop through multiple sequential stages, each associated with characteristic phenotypes: initial attachment, irreversible attachment, maturation, and dispersal. These all represent the fundamental constituents of the biofilm formation process [11]. Once a foreign body enters the body, the host extracellular matrix (ECM) quickly coats the new material: bacteria (especially *S. Epidermidis* and *S. Aureus)* express proteins, during their growth phase, which form strong interactions with the host surfaces, resurfacing the ECM; this bonding to the foreign surface becomes quickly irreversible, making it very difficult, for host antibodies and for delivered antibiotics, to disrupt it [11].

Modern TJA implants consist of a mixture of titanium, cobalt-chrome, ceramic (hip arthroplasty) and vitamin-E-stabilized ultra-high-molecular-weight polyethylene (UHMWPE), which has shown improved mechanical strength compared to irradiated and melted UHMWPE and a promising capacity to limit the bacterial adhesion on TJA implants, reducing PJI risk [12]. 

Several nanoparticles have been shown to have passive antimicrobial capacities: silver, copper, quantum dots and zinc oxide are excellent examples. Although bacterial growth has been shown on titanium and cobalt-chrome [13], the surface properties could be modified in order to prevent bacterial adhesion. Silver nanoparticles (nanosilver; 5 ± 2 nm) have been shown to reduce cell viability thanks to their ability to enter the intracellular space without interfering with the osteogenic process necessary for bone ongrowth over the implant [14]. Nanosilver coatings have been extensively studied and the results are very promising: several nanosilver formulations allow for a slow solubilization of silver over an extended period of time, without excessive, local increases in silver concentration leading to cytotoxicity towards osteoblasts [15]. Nanosilver is also active against *E. coli* and *S. aureus* [16], has anticorrosive characteristics [17] and is easily incorporated into hydroxyapatite and chitosan to reproduce an antibacterial coating with osteo-integrative capability [18]. However, current methods of nanosilver synthesis are based on the use of organic solvents and stabilizers that are potentially toxic to the environment, and, thus, the green synthesis of silver nanoparticles should become a prerequisite for the development and application processes of nanotechnology. New synthetic methods have been recently proposed in order to increase the biological safety of nanosilver: supercritical CO_2,_ starch, glucose, maltose and gelatin have been all used as reductants and stabilizers to prepare nanosilver [19,20,21].

### 2.2. Nanoparticles for Antibiotic Delivery

Prophylactic systemic antibiotics are routinely administered to patients undergoing total joint arthroplasty to prevent a postoperative PJI. However, this clinical practice is limited by multiple disadvantages: increasing antibiotic resistance, duration of the prophylaxis, correct timing in relation to the surgery, the usual low concentration at the target site and the well-known inability of antibiotics to disrupt biofilms [3].

Antimicrobial nanoparticles have been engineered in order to prevent infections in orthopedic surgery [22]. Poly L-lactic acid (PLLA) nanofiber scaffolds have been engineered into bio-absorbable scaffolds for tissue regeneration: the addition of poly lactic-co-glycolic acid (PLGA) nanospheres increased the extended release of doxycycline [23]. PLLA and PLGA scaffolds have been successfully used, in the animal model, as a delivery system of multiple antibiotics (gentamicin, rifampin, vancomycin) in order to prevent osteomyelitis [24]. Nanophase selenium represents another promising nanomaterial able to prevent musculoskeletal infections as a collateral effect of its chemotherapeutic applications [25].

## 3. Periprosthetic Joint Infection (PJI) Treatment

The recent literature from the International Consensus Meeting on Periprosthetic Joint Infections (ICM 2018) [5] has confirmed that many contemporary conservative measures failed to eradicate the biofilm from the periprosthetic environment. A recent metanalysis from Horriat and et al. [26] showed that, following the appropriate preoperative indications, Debridement, Antibiotic, Implant Retention (DAIR) has an average success rate of 66%. Calanna et. al. [27] reported a success rate of 80% when an accurate debridement followed by the use of antibiotic-added calcium sulphate beads were utilized to treat acute PJI: this last procedure is characterized by thermal (Argon bean) and mechanical (chlorhexidine sponge) aggression towards the biofilm, which, as previously described, strongly bonds to the implant surface. 

The contemporaneous, North American “gold standard” treatment for chronic PJI is still represented by a two-stage revision: a temporary polymethylmethacrylate cement spacer that elutes a high concentration of antibiotics is placed into the joint at the time of component explant [28]. The use of cement spacers has several limitations. First, the mechanical properties of current cement spacers are very poor and their use has been associated with joint dislocation, fracture of the construct and major peri-articular bone loss [29]. Additionally, the elution of antibiotics from the spacers, at concentrations above the Minimum Inhibitory Concentrations (MIC), is very limited in time (72 h) [30], and, unfortunately, acute postoperative renal injury has been reported during the peak of antibiotic release from the spacer [31]. Another major limitation of the use of cement spacers is represented by the finding that a bacterial biofilm has been detected on their surfaces at the time of revision surgery [32]. This finding jeopardizes the notion of sterility of the intra-articular space at the time of the re-implant. 

For these reasons, two-stage revision following chronic PJI has been shown to have a 14.4% mortality rate and extremely high morbidity [33]. This dramatic finding has necessitated novel methods of eradicating biofilm formation from TJA components. Nanoparticles and nanotechnology represent very appealing methods to achieve this goal: they are highly biocompatible and they have the capability of delivering both hydrophilic as well as hydrophobic drugs without the toxicity related to antibiotic boluses [34]. 

Multiple efforts to improve the structural and anti-biofilm characteristics of antibiotic-added cement spacers are underway. Gillani et al. [8] extensively studied the addition of ceramic particles (barium sulfate and zirconia) to bone cement. Interestingly, they demonstrated an increase in cytocompatibility and a decrease in mechanical failure of the cement under stress conditions. Lipid nanoparticles, nano-silica and clay nanotubes have been recently added to standard bone cement (PMMA or polymethylmethacrylate) to enhance drug delivery in a prolonged fashion [35,36,37]. 

Various nanoparticles have been studied as intra-articular antibiotic carriers, including solid lipid nanoparticles (50–100 nm lipid colloidal carrier), polymeric micelles (10–1000 nm in size) and, ultimately, liposomes, which are phospholipid vesicles between 20 and 100 nm in size. Interestingly, nanoparticles have demonstrated the capability of enhancing antibiotic penetration within a well-matured biofilm in multiple in vitro studies: multiple interaction mechanisms between nanoparticles and viable bacterial cells have been described [38,39,40], all resulting in bacterial cell death within the biofilm. Unfortunately, in vivo studies are limited and it is unclear how antibiotic-delivery nanoparticles interact with biofilms in vivo. Li et al. [41] were the first to encapsulate antibiotic (daptomycin) in liposomes to inhibit *S. aureus* biofilm growth in a mouse model, supporting the idea of using nanoparticles for the effective delivery of antibiotics into a mature biofilm. Unfortunately, the clinical use of liposomal or polymeric nanoparticle delivery systems has several potential drawbacks: high cost of production, uncertainty in drug loading and bonding and possible general toxicity of the construct.

Several metal nanoparticles have shown direct bactericidal effects against well-formed biofilm: silver, copper, gold, titanium and zinc all have intracellular, antimicrobial properties. In particular, silver ions released from nanoparticles can generate reactive O_2,_ leading to disruption of cell membrane integrity and inhibition of cell proliferation [42]. Nevertheless, the direct bactericidal potential of nanoparticles still lacks direct, repetitive in vivo testing. 

The current literature has paid particular attention to many derivates of graphene [43], such as multilayer graphene, graphene oxide (GO) and reduced graphene oxide (rGO), because of their mechanical and antimicrobial properties. In fact, GO materials have intrinsic antibacterial properties, in combination with anti-adhesion and contact-killing properties [43]. On the other hand, the durability of the GO coating and the different levels of sensitivity of bacteria to the GO antibacterial effect have to be established. 

Recently, the use of nanotechnology and nanoparticles for energy conduits from external sources (lasers, alternative magnetic field—AMG) has been proposed as a methodology for biofilm disruption. In fact, Kim et al. [44] demonstrated that increasing the nanoparticle temperature induces irreversible thermal damage to the bacterial cells living in biofilms. In a similar study, Meeker et al. used antibiotic-loaded, spherical gold nanoparticles to disrupt *S. aureus* and *P. aeruginosa* biofilms. Interestingly, they were able to activate the release of antibiotics directly to the mature biofilm thanks to the use of pulse laser irradiation. Magnetic nanoparticles represent another option for the induction of thermal damage in the biofilm: magnetic nanoparticle (i.e., iron oxide) heating effectively disrupts biofilms of *S. aureus* and other microorganisms [45,46]. Unfortunately, the clinical translation of these thermal technologies has been limited by the risk of harming adjacent and even more distant tissues. 

## 4. Conclusions

Periprosthetic joint infection (PJI) represents a devastating complication of total joint arthroplasty with an extremely high rate of morbidity and mortality. The current, invasive treatments lack the ability to prevent biofilm formation and to eradicate mature biofilms. 

Nanomaterials have recently exhibited major advantages over classical orthopedic materials and treatment strategies because of their high biocompatibility and bioreactivity. The recent literature has shown that changes in the properties of material at the nanoscale have the possibility to directly interfere with the development of biofilms. This novel treatment has the potential to optimize TJA component surfaces to prevent bacterial biofilm formation and provide prolonged, local delivery of antibiotics to eradicate already established planktonic bacteria. Nevertheless, the full role of physicochemical properties of nanoparticles in the biological systems of the human body needs to be addressed. Further investigations are needed to fully understand the potential, and especially the safety, of this exciting nanotechnology. 

## Data Availability

All underlying data are in the text.
